# William Edward Sevier Burnett

**Published:** 1910-05-28

**Authors:** 


The death is announced of William Edward Sevier Burnett, Esq., L.R.C.P., M.R.C.S., of Fairlie, Bowdon, Cheshire, at the age of seventy years. He was a Dublin student, and took his L.M. and L.A.H. Dublin in 1867, and two years later the Edinburgh Conjoint Diploma.

